# Development of a Bayesian Classifier for Breast Cancer Risk Stratification: A Feasibility Study

**Published:** 2010-03-29

**Authors:** Alexander Stojadinovic, Christina Eberhardt, Leonard Henry, John Eberhardt, Eric A. Elster, George E. Peoples, Aviram Nissan, Craig D. Shriver

**Affiliations:** ^a^Department of Surgery, Division of Surgical Oncology, Walter Reed Army Medical Center, Washington, DC; ^b^United States Military Cancer Institute, Clinical Trials Group, Washington, DC; ^c^National Naval Medical Center, Bethesda, MD; ^d^DecisionQ Corporation, Washington, DC; ^e^Brooke Army Medical Center, Fort Sam Houston, TX; ^f^Hadassah Hebrew University Medical Center, Mount Scopus, Jerusalem, Israel

## Abstract

**Background:** Lifetime risk assessment tools are relatively limited in identifying breast cancer risk in younger women. The predictive value of mathematical models to estimate risk varies according to age, menopausal status, race/ethnicity, and family history. Current risk prediction models estimate population, *not* individual, levels of breast cancer risk; hence, individualized risk prediction models are needed to identify younger at-risk women who could benefit from timely risk reduction interventions. Clinical data collected as part of breast cancer screening studies may be modeled using Bayesian classification. **Purpose:** To train a proof-of-concept Bayesian classifier for breast cancer risk stratification. **Patients and Methods:** We trained a Bayesian belief network (BBN) model on cohort data (including risk factors, demographic, electrical impedance scanning (EIS), breast imaging, and biopsy data) from a prospective pilot screening trial in younger women (*N* = 591). Receiver operating characteristic curve analysis and cross-validation of the model were used to derive preliminary guidance on the robustness of this approach and to gain insights into what a cross-validation exercise could provide in terms of risk stratification in a larger population. **Results:** Independent predictors of biopsy outcome in the BBN model included personal breast disease history, breast size, EIS (low vs high risk) and imaging results, and Gail cutoff (5-year risk: <1.66% vs ≥1.66%). Area under the receiver operating characteristic curve and positive predictive value for benign and malignant biopsy outcomes were 0.88 and 97% and 0.97 and 42%, respectively. Patient-specific probability of biopsy outcome given positive EIS result and Gail model 5-year risk ≥1.66% indicated that the combined effect of these predictors on likelihood that a biopsy would prove malignant exceeded the sum of the individual effects; breast cancer likelihood is as follows: 3% (EIS negative and Gail model 5-year risk <1.66%) versus 9% (EIS positive and Gail model 5-year risk <1.66%) versus 27% (EIS negative and Gail model 5-year risk ≥1.66%) versus 45% (EIS positive and Gail model 5-year risk ≥1.66%). **Conclusion:** Clinical data collected as part of breast cancer screening studies can be modeled using Bayesian classification. The BBN model may be predictive and may provide clinically useful incremental risk information for individualized breast cancer risk assessment in younger women.

Breast carcinoma is the most commonly diagnosed cancer and the second leading cause of cancer-related mortality among women in the United States.^1^ In 2009, there were more than 192000 estimated new cases of cancer of the breast and more than 40000 disease-specific deaths.^1^ Breast cancer—related mortality rates have steadily decreased over the past 2 decades, largely because of improved disease detection and therapy.^2^

As breast cancer in younger (age < 40 years) women is infrequently diagnosed in the early stages by utilizing current screening guidelines, improved cancer screening and detection methods are important in current research, particularly in younger, at-risk women.^3^ Breast cancer in younger women typically has unfavorable prognostic characteristics associated with increased disease-specific mortality; hence, early detection in younger women is imperative.^4^^-^6 Younger women are not referred for periodic imaging unless they are identified as being “high risk.”^7^ “At risk” younger women with significant family history or genetic factors are encouraged to undergo frequent clinical and annual breast imaging surveillance and to consider chemoprevention.

While increased surveillance for at-risk women may be beneficial, the value of this approach is restricted by the rarity of breast cancer due to known genetic risk factors.^8^,^9^ More than 90% of breast cancers are detected in women who are *not* identified as being high risk.^3^ Furthermore, screening mammography is generally less accurate in younger women and those with increased breast tissue density commonly encountered in women younger than 40 years.^10^ The reduced sensitivity of mammography for dense breasts impacts age groups in which a “life saved” often results in “higher” personal and societal costs in terms of altered life expectancy and personal productivity.^11^

Magnetic resonance imaging (MRI) is being used increasingly as a screening modality in high-risk women with a significant family history of breast cancer or those with *BRCA1* or *BRCA2* gene mutations, resulting in lifetime risk of cancer exceeding 20%.^12^,^13^ Hence, breast MRI is currently applied to a relatively small proportion of all women. Because MRI is unaffected by breast tissue density, it is appealing to consider its use for screening young women in general; however, the high cost, requirement for intravenous contrast administration, and variable specificity limit its feasibility for widespread population-based screening.^14^,^15^ Therefore, improved methods for risk prediction in younger women are needed to identify those at high risk for breast cancer.

Tamoxifen may be considered in both premenopausal and postmenopausal women, and raloxifene may be considered in postmenopausal women, with lobular carcinoma in situ or with a 5-year breast cancer risk estimate of 1.66% or higher (according to the Gail model or the National Cancer Institute Breast Cancer Risk Assessment Tool), in order to reduce the risk of estrogen receptor-positive breast cancer.^16^ In the National Surgical Adjuvant Breast and Bowel Project (NSABP) P-1 study tamoxifen (20 mg/day for 5 years) consistently reduced the incidence of breast cancer by 49% in at-risk women across all study age and risk groups (women age 35—59 with a ≥1.66% risk, those 60 years or older, or with those prior LCIS) thereby demonstrating the efficacy of chemoprevention for this disease.^17^ The Multiple Outcomes of Raloxifene Evaluation (MORE), Continuing Outcomes Relevant to Evista (CORE), Raloxifene Use for the Heart (RUTH,) and NSABP Study of Tamoxifen and Raloxifene (STAR) trials demonstrated consistent significant reductions in estrogen receptor-positive breast cancer incidence in at-risk postmenopausal women.^16^ Subsequent analyses of the NSABP P-1 study data suggested improved quality-adjusted survival and cost-effectiveness when tamoxifen was initiated as early as age 35 years in at-risk (Gail model 5-year risk ≥1.66%) women.^18^,^19^ Hence, identification of women who are at high risk and may benefit from chemoprevention is of particular importance.

Lifetime relative risk assessment tools (eg, Gail model) are available to identify women older than 35 years who are at risk for breast cancer. However, the predictive value of mathematical models to estimate breast cancer risk varies according to age, menopausal status, race/ethnicity, and family history of breast cancer. Instruments such as the Gail model are imperfect for identifying increased cancer risk in younger women.^20^ Importantly, all current risk prediction models estimate population, *not* individual, levels of breast cancer risk. Currently, the only criterion generally used to identify high-risk young women who could benefit from chemoprevention is family/genetic history. The value of this risk estimation paradigm is limited by the rarity of breast cancer due to known gene mutations. Better individualized risk prediction models are needed to identify younger at-risk women who could benefit from risk reduction interventions and earlier chemoprevention.

Bayesian belief network (BBN) models have been used in research to better understand research data and biologic systems such as functional genomics. In recent years, these applications have been applied to better understand clinical problems, such as models developed to estimate breast cancer risk in mammographic microcalcifications and predict false-positive mammograms.^21^,^22^ We believe that clinical data collected as part of breast cancer screening studies may be modeled using Bayesian classification. The objective of this study was to train a proof-of-concept machine-learned BBN model based on previously unpublished cohort data from this prospective pilot screening trial and to perform cross-validation for the purposes of evaluating the feasibility of using readily available data (including risk factors, demographic data, breast impedance, and breast imaging) and the Bayesian classification for breast cancer risk stratification (estimating biopsy outcome) in younger women.

## METHODS

### Patients

Between August 2002 and March 2005, a total of 591 female military healthcare beneficiaries were enrolled into this institution review board—approved, single-arm, prospective pilot screening trial. The clinical protocol was reviewed and approved by institutional review boards of the Walter Reed Army Medical Center, Washington, DC, and the Keller Army Hospital, West Point, NY. Subjects who met the eligibility criteria were offered participation in this study. Subjects were recruited from the gynecology clinic or the Comprehensive Breast Center at Walter Reed Army Medical Center or the gynecology or family practice clinic at Keller Army Hospital. Study inclusion criteria consisted of younger women aged 18 to 49 years who provided informed consent prior to study enrollment and who were willing to be followed at the participating institution. Age was stratified for analysis as follows: younger than 30, 30 to 34, 35 to 39, and 40 to 49. Potential study subjects were excluded if they had breast surgery (including core biopsy) or were lactating within the preceding 3 months, had breast fine needle aspiration within the preceding 1 month, were pregnant, had electrically powered implanted devices (eg, pacemaker), or were undergoing chemotherapy or radiation treatment. Data collected for each study subject included age, race/ethnicity, clinical history (personal and family history of breast cancer, previous breast surgery or biopsy, and results of those interventions), hormonal information (age of menarche and first full-term pregnancy, menstrual status, date of last menstrual period, and exogenous hormone use), breast density and size (bra cup size), Gail model risk estimate, results of clinical breast examination (CBE), screening breast electrical impedance scanning (EIS), conventional imaging, and biopsy results. All study participants underwent EIS of the breast by using the T-ScanTM 2000ED (Mirabel Medical, Austin, TX) as previously described.^23^

### Statistical Methods

The BBN model was trained by using a priori variables to estimate the likely diagnostic outcome of breast biopsy. The BBN model was developed by using commercially available machine-learning algorithms (FasterAnalytics, DecisionQ, Washington, DC), which automatically learn network structures and joint probabilities from the prior probabilities in the data. BBN models are a type of directed acyclic graph, which means that they represent information in a hierarchical format. BBN models allow us to identify those variables that contain the most information and are thus most useful for estimating outcomes. The associations represented by BBN models are associations of conditional dependence, allowing us to estimate the posterior likelihood of a given outcome given prior observations.

In order to refine the model, a stepwise training process was used. Quantitative and qualitative assessments were used to optimize variable preparation and selection in order to produce the most robust and useful model. The objective was to produce the optimum biopsy outcome estimate through iterative quality assurance and reduction of confounding information. This process used to develop the model is summarized as follows: (1) preliminary modeling to identify appropriate machine-learning parameters, data quality issues, and confounding features and feature analogues that reduce model accuracy, (2) global modeling to set appropriate machine-learning parameters, remove identified analogs and confounders, and perform full “queue learning” to observe global data structure, (3) naive modeling of the outcome of interest to identify the relative contribution of covariates, and (4) focused modeling by using “queue learning” on subsets of variables identified in the prior steps to derive a more focused BBN model than that obtained in global modeling. By excluding marginal or noncontributory variables, the remaining ones are explored more exhaustively.

Cross-validation was performed on the final focused Bayesian classifier by using a train-and-test cross-validation methodology to produce classification accuracy estimates. Fivefold cross-validation was performed by randomizing the data set into 5 separate and unique train-and-test sets. Each set consists of a training set composed of 90% of patient records and a test set consisting of the remaining 10% of records. Once the model was constructed with a training set, the matching test set was entered into the model, generating a case-specific prediction for each record for independent variables of interest. A receiver operating characteristic curve was plotted for each test to calculate classification accuracy. The receiver operating characteristic curve was used to calculate area under the curve, a metric of overall model quality, and to calculate corresponding predictive values for biopsy outcome.

## RESULTS

The study population comprised an ethnically diverse group of younger women (41% non-Caucasian), healthcare beneficiaries in a free access system of military medical care. Of the 591 study participants, 67% were younger than 40 years (mean age: 35 ± 6.9 years) and 90% were premenopausal (Table 1). Two percent of the study population was taking exogenous hormones at the time of study enrollment; however, there was no statistically significant association with disease (*P* = .95). Fifty-five percent of participants had no family history of breast cancer, and family history was only marginally associated with biopsy outcome (*P* =.10). The findings of CBE were statistically associated with both age (*P* =.01) and disease (*P* ≤ .001); 31% of subjects had findings that were deemed not suspicious, whereas 4% of subjects had suspicious CBE findings. Five percent of study subjects had an estimated 5-year risk of breast cancer ≥1.66% according to the Gail model, and these findings were statistically associated with both disease and age of subject (*P* ≤ .001). Mammography was performed in 281 women and was found to be Breast Imaging Reporting and Data System (BIRADS) III or higher in 75 cases (27%); mammography was found to be statistically associated with both disease and age of subject (*P* ≤ .001). Breast ultrasound examination was performed in 258 women and was found to be BIRADS III or higher in 66 cases (26%); ultrasound was found to be statistically associated with disease (*P* ≤ .001) but not with age (*P* = .18).

We also studied other well-known risk factors in our population and identified 3 risk factors that were not statistically associated with biopsy outcome: mean age at menarche (*P* = .12), mean age at first pregnancy (*P* = .39), and nulliparity (*P* = .93). Finally, there was no statistically significant difference between the mean age of our population (35 years) and the mean age at time of cancer diagnosis (38 years, *P* = .35) or diagnosis of premalignant histopathology (38 years, *P* = .56). We tabulated data by age group and biopsy outcome as shown in Tables 1 and 2.

Of the 591 women enrolled in the study, 568 were found to be EIS negative (low risk) and 23 were found to be EIS positive (high risk). In the EIS-negative group, 95 underwent biopsy and 87 were benign on final histopathology. The remaining 8 were either premalignant (*n* = 4) or malignant (*n* = 4). In the EIS-positive group, 10 underwent biopsy; 5 were benign, whereas 5 were either premalignant (*n* = 3) or malignant (*n* = 2). Of 13 premalignant or malignant lesions, EIS identified 5 (38.5%). The negative predictive value (NPV) of the EIS-negative group was 92%, whereas the positive predictive value (PPV) of the EIS-positive group was 50%.

**Figure 1:**
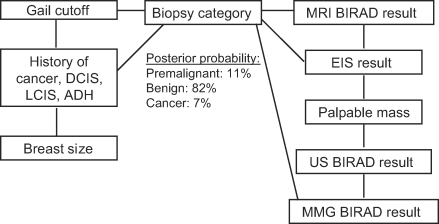
Exemplar BBN model of breast biopsy outcome with expected frequency of histology in our study population. BBN indicates Bayesian belief network; LCIS, lobular carcinoma in situ; MRI, magnetic resonance imaging; BIRAD, Breast Imaging Reporting and Data System; EIS, electrical impedance scanning; US, ultrasound; MMG, mammography; DCIS, ductal carcinoma in situ; and ADH, atypical ductal hyperplasia.

We trained a proof-of-concept BBN model on this pilot cohort data and performed analysis and cross-validation. The Bayesian Network shown in Figure 1 indicates that the six nearest independent associated features (direct relationship to breast biopsy diagnosis) used to estimate a breast biopsy diagnosis (Biopsy category) are screening breast EIS result, Gail model cutoff (5-year risk estimate <1.66% vs ≥1.66%), mammogram BIRAD result, MRI BIRAD result, breast size, and personal history of breast disease. This does not mean, however, that “Any Palpable Mass” on CBE and ultrasound BIRAD results (indirect relationship to breast biopsy diagnosis—Fig 1) do not influence the estimate of likely biopsy diagnosis, but rather that they are conditionally independent of biopsy outcome, given knowledge of screening breast EIS and MMG BIRAD results.

The BBN feasibility model was validated using train-and-test cross-validation and produced strongly predictive areas under the curve (0.75—0.97) for differentiating malignancy and premalignant disease from benign findings (Table 3). Cross-validation also produces a 97% NPV and a 42% PPV for malignancy. It is important to note that with a relatively small set of outcomes, there is a high degree of variance in results between cross-validation exercises (Table 3). The BBN model is a recursive information structure, and the inclusion of conditional dependence between predictive variables guards against overinterpretation of data (overfitting). The model informs estimates not only through estimation of biopsy outcome but also through estimation of as-yet-unknown imaging results, wherein estimates of biopsy outcome can be derived from available clinical and imaging data, even if some imaging studies are unavailable at the time of biopsy outcome estimation.

**Figure 2:**
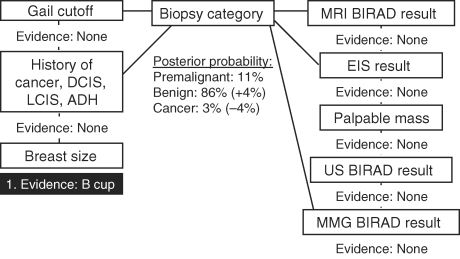
Probability of cancer diagnosis given only evidence of breast size (bra cup) B is 3%. LCIS indicates lobular carcinoma in situ; MRI, magnetic resonance imaging; BIRAD, Breast Imaging Reporting and Data System; EIS, electrical impedance scanning; US, ultrasound; MMG, mammography; DCIS, ductal carcinoma in situ; and ADH, atypical ductal hyperplasia.

**Figure 3:**
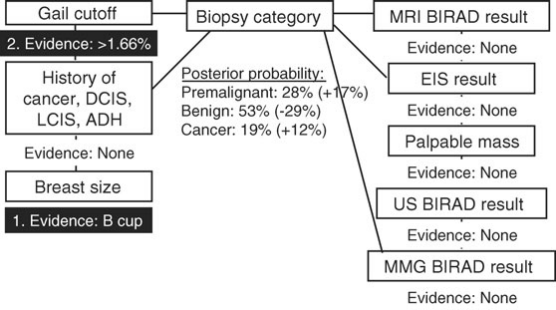
Probability of cancer diagnosis given only evidence of bra cup size B and Gail model 5-year risk ≥1.66% is 19%. LCIS indicates lobular carcinoma in situ; MRI, magnetic resonance imaging; BIRAD, Breast Imaging Reporting and Data System; EIS, electrical impedance scanning; US, ultrasound; MMG, mammography; DCIS, ductal carcinoma in situ; and ADH, atypical ductal hyperplasia.

**Figure 4:**
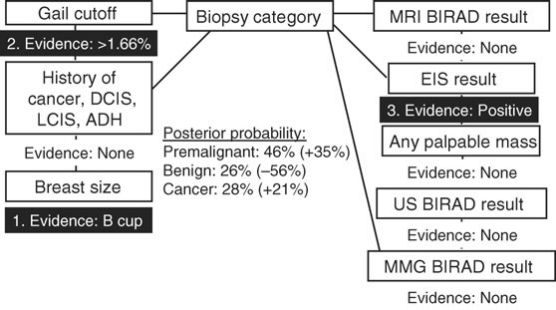
Probability of cancer diagnosis given only evidence of bra cup size B, Gail model 5-year risk ≥1.66%, and EIS positive result is 28%. EIS indicates electrical impedance scanning; LCIS, lobular carcinoma in situ; MRI, magnetic resonance imaging; BIRAD, Breast Imaging Reporting and Data System; US, ultrasound; MMG, mammography; DCIS, ductal carcinoma in situ; and ADH, atypical ductal hyperplasia.

To demonstrate the use of this type of model, we walk through an example case of how we can use available information to estimate clinically relevant outcomes using the network. Knowledge of breast size (bra cup) B (Fig 2: Evidence 1) results in slightly lower risk of cancerous biopsy result (-3.6%) for the subject compared with our reference population. When the additional knowledge of Gail model 5-year high-risk estimate (Fig 3: Evidence 2) is added to refine the posterior estimate of biopsy outcome given previously known breast size (bra cup) B, there is a 12% increased likelihood of cancerous biopsy and a 17% increase in the likelihood of premalignant histology, relative to our overall study cohort. Finally, adding knowledge of a positive (high risk) EIS screening result (Fig 4: Evidence 3) increases the posterior risk estimate of cancerous biopsy by 21% and the risk estimate of premalignant disease by 35%. Each posterior probability estimate is the result of adding evidence for each factor being used to make the estimate. As new evidence is “added” for a given factor, the existing evidence already input remains unchanged. However, other nodes in the network for which no evidence is available have their posterior probabilities updated given other evidence that has been input, and these new posteriors in turn influence the predicted variable.

As all features in the model are, at some level, conditionally dependent with biopsy outcome, those features available at the time of initial clinical visit (a priori knowledge) can be selected and applied to the model to estimate biopsy outcome. Subsets of features can also be used to generate an inference table (Table 4) that can be used to quickly estimate biopsy outcome for all known combinations of the identified features. The incremental values of both screening breast EIS and the Gail model data are shown in the inference table, Table 4. Under the most favorable circumstances (EIS negative and Gail model 5-year risk <1.66%) the risk of malignancy is 3%, and under the least favorable circumstances (screening EIS positive and Gail model 5-year risk ≥1.66%), the risk of malignancy is 45%.

## DISCUSSION

For the majority of younger women, namely those considered to be average risk for developing breast cancer under the current risk assessment and screening model, the only generally available risk assessment modality is CBE, which is imperfect as a screening tool as it has an unacceptably low sensitivity and high false-positive rate compared with mammography.^24^ CBE detects cancers only when they have advanced to the point of being palpable. When cancers are clinically palpable, they have reached a more advanced stage of disease. Palpable breast cancers typically require more aggressive and costly treatments, with concomitant worse quality of life and oncological outcomes.^25^,^26^

The early detection of breast cancer in younger women is very important, particularly because it demonstrates aggressive tumor biology with rapid tumor growth, demonstrates a relatively short preclinical disease phase, and has worse cancer-specific survival than in older women.^6^,^27^,^28^ A risk stratification paradigm that improves upon the interpretation of existing clinical information should allow us to detect disease at an earlier stage of development. The Gail model offers an improvement in predicting risk, yet it is still an imperfect tool because it is designed using a primarily older, Caucasian population. Further, as more effective personalized detection, prevention, and treatment strategies become available for breast cancer in younger women, strategies and technologies that support truly personalized risk assessment and screening can favorably impact survival, especially if conducted at shorter intervals than in older women.^28^^-^33

Recognizing the need for individualized breast cancer risk assessment tools for younger women, we conducted a feasibility study to determine whether a machine-learned BBN model could be developed to support individualized breast cancer risk stratification. The model trained and cross-validated in this study was based on data from a prospective pilot screening trial in younger women (*N* = 591) and produced receiver operating characteristic curves, when cross-validated, with areas under the curve of 0.88, 0.97 and 0.75 for benign, malignant, and premalignant findings, respectively. This proof-of-principle study shows that clinical data collected as part of routine, current breast cancer screening studies can be developed into enhanced screening tools with improved sensitivity and specificity by using machine-learned BBN models. These networks can use readily available information to estimate clinically relevant outcomes, providing clinically useful incremental risk information for individualized breast cancer risk assessment in younger women.

In our BBN model, the features showing direct conditional dependence with biopsy outcome include (1) personal history of breast disease, (2) breast size (bra cup). (3) EIS (low vs high risk), (4) breast imaging results, and (5) Gail model risk cutoff (5-year risk <1.66 vs ≥1.66). Each of these variables is also significantly related to biopsy category when examined using the calculated χ^2^. CBE and breast ultrasound results were also determined to be statistically associated with biopsy outcome, and the Bayesian model includes these features as well, but they are associated with biopsy outcome through EIS and mammography results. Other features that showed statistical significance, but dropped out of the training process in the model, include patient ethnicity, menopausal status, and prior breast biopsy. The machine-learning process is designed to produce a simpler (parsimonious) model whenever possible; thus, these additional factors were likely surpassed by more specific imaging and personal history risk factors.

Interestingly, a number of attributes were found to have statistically significant association with patient age, including CBE findings, mammography BIRADS category, nulliparity, and Gail model 5-year risk score. Conversely, certain factors found to be significantly associated with biopsy outcome, using both bivariate statistical tests and the machine-learning process, were not associated with patient age: breast (bra cup) size, EIS screening examination, or MRI. There was no statistically significant difference in mean age at diagnosis of premalignant or malignant disease compared with the mean age of our study population, and when biopsy results were examined by age category, they still did not demonstrate any statistically significant associations. Finally, features considered as well-established breast cancer risk factors in the general population were not found to have statistically significant associations with biopsy outcome in our younger population, including family history of breast cancer, age at menarche, nulliparity, and age at first pregnancy. While these are considered as common risk factors for developing breast cancer, data suggest that these variables are not effective to determine individual risk of cancerous or premalignant lesions in our study population. Although we must be cautious not to overinterpret these findings, they do raise important questions about the appropriate risk measures in a younger population of ethnically diverse women when well-established risk factors have statistically significant association with subjects' age while our disease outcome appears to be age independent.

Having trained, encoded, and validated the machine-learned BBN model, we can estimate the likely biopsy outcome given readily available clinical and imaging data. However, the BBN model not only allows the posterior estimation of the likely biopsy outcome but also identifies a hierarchy of conditional dependence that allows us to identify which pieces of information are most useful in calculating our estimate. This hierarchy also defines how independent variables influencing biopsy outcome also influence one another, providing a better understanding of how the estimate is derived and providing an opportunity to estimate missing parameters by using those currently available for any given patient. It is notable that the combined effect of these independent predictors on the likelihood of disease is greater than the sum of the individual effects. By way of example, mammography finding of BIRAD IV increases the likelihood of a malignant biopsy result in our study population by 5%, whereas a Gail model 5-year risk score of greater than 1.66% increases the likelihood of malignancy by 26%, yet together these findings increase the likelihood of disease by 42%—greater than the sum of their individual effects.

Importantly, the current proof-of-principle model estimates probability of having a breast abnormality biopsied and having it show the underlying malignancy. The ultimate clinical utility of such a model with appropriate sample size, population disease incidence, and follow-up will be based on robust predictive value of the model for developing breast cancer. The most clinically relevant model will be based on easily obtainable nonimaging parameters to identify at-risk women, who could benefit from breast imaging—based screening and risk reduction interventions.

## CONCLUSION

A need exists for a breast cancer risk estimation paradigm that can be used along with relevant demographic, clinical, and other readily obtainable patient-specific data in younger women in order to provide an individualized cancer risk assessment, direct screening efforts that can lead to prophylaxis, and detect breast cancer at an early stage. The computational complexity of designing such risk stratification algorithms for the average-risk woman necessitates a large, multidimensional cohort and requires a selection and encoding methodology that is both robust and transparent so as to sustain clinical scrutiny and improve clinical practice. For our study, we integrated multidimensional clinical, imaging, and pathological data from a prospective cohort to test the feasibility of model development. Using this cohort, we trained a BBN model by using machine-learning algorithms and developed a risk classification model for the average-risk younger woman, with promising cross-validation results. Our proof-of-concept study shows that this type of model could be used to perform individualized screening on a regular basis, using available clinical data at low cost, and that it warrants further assessment and independent testing.

## Acknowledgment

The authors thank Tiffany Felix for her invaluable assistance supported in part by the Henry M. Jackson Foundation for the Advancement of Military Medicine and the members and staff of the United States Military Cancer Institute and the Clinical Breast Care Project for their consistent support of this collaborative research effort.

## Figures and Tables

**Table 1:** Summary of study population characteristics by age*

	Age category, y				
Characteristic	<30	30-34	35-39	40-49	*P*
Menopausal status					<.001
Premenopausal	124	114	122	171	
Postmenopausal	0	5	15	34	
Perimenopausal	0	0	1	4	
Not recorded	0	0	1	0	
Screening breast EIS result					.4515
Negative	120	117	132	199	
Positive	4	2	7	10	
Clinical breast examination result					.0077
No findings	78	88	77	141	
Not suspicious	45	28	52	57	
Suspicious	1	3	10	11	
Hormone replacement therapy					.0263
Current	0	2	2	11	
Past	0	1	0	3	
Never	124	116	137	195	
Mammogram results					<.001
BIRADS 0	1	0	3	10	
BIRADS I or II	6	16	61	109	
BIRADS III	1	6	8	21	
BIRADS IV	0	2	10	23	
BIRADS V	0	1	2	1	
No mammogram	116	94	55	45	
Breast biopsy category					.4076
Benign, no atypia	19	12	27	34	
Premalignant	1	0	2	4	
Infiltrating cancer or DCIS	0	1	2	3	
No biopsy (assumed benign)	104	106	108	168	
Family history category					.4080
One first degree	9	12	20	18	
One second degree	22	24	24	38	
One first and one or more second degree	9	9	12	11	
Two or more first degree	0	1	4	2	
Two or more second degree	7	12	10	21	
No significant family history	77	61	69	119	
Gail model 5-year risk category, %					<.001
<1.66	108	119	132	175	
≥1.66	0	0	3	27	

*BIRADS indicates Breast Imaging Reporting and Data System; EIS, electrical impedance scanning; and ductal carcinoma in situ.

**Table 2:** Summary of study population characteristics by biopsy category*

	Biopsy category				
Characteristic	Benign, no atypia	Infiltrating cancer or DCIS	Premalignant	No biopsy (benign)	*P*
Mean age at menarche, y†	13	12	13	13	.9997
Mean age at first pregnancy, y†	25	22	24	24	.8622
% Nulliparous	79.1	100.0	66.7	75.8	.9299
Mean age at diagnosis, y†	36	38	38	35	.0959
Menopausal status					.0307
Premenopausal	86	6	6	433	
Postmenopausal	6	0	0	48	
Perimenopausal	0	0	1	4	
Screening breast EIS result					<.001
Negative	87	4	4	473	
Positive	5	2	3	13	
Clinical breast examination result					<.001
No findings	19	2	4	359	
Not suspicious	55	1	2	124	
Suspicious	18	3	1	3	
Hormone replacement therapy					.9460
Current	1	0	0	14	
Past	1	0	0	3	
Never	90	6	7	469	
Bra cup size					.0094
A	4	3	0	39	
B	25	1	3	123	
C	18	0	1	110	
D+	6	1	1	65	
Not recorded	39	1	2	149	
Mammogram results					<.001
BIRADS 0	3	0	3	8	
BIRADS I or II	27	0	2	163	
BIRADS III	6	0	0	30	
BIRADS IV	26	3	2	4	
BIRADS V	0	3	0	1	
No mammogram	30	0	0	280	
Family history category					.1035
One first degree	7	1	0	51	
One second degree	18	1	2	87	
One first degree and one or more second degree	0	1	0	6	
Two or more first degree	6	0	2	42	
Two or more second degree	55	2	3	266	
No significant family history	6	1	0	34	
Gail model 5-year risk category, %					<.001
<1.66	86	2	5	441	
≥1.66	4	3	1	22	
Not recorded	2	1	1	23	

*BIRADS indicates Breast Imaging Reporting and Data System; EIS, electrical impedance scanning; and DCIS, ductal carcinoma in situ.

†*P* value of cancer and premalignant populations compared with benign population.

**Table 3:** Feasibility model cross-validation statistics

Area under the curve			Predictive value, %		
	Benign	Cancer	Premalignant	Benign	Cancer	Premalignant
Internal	0.98	0.99	0.97	100.0	66.7	50.0
Exercise 1	0.94	0.98	0.58	100.0	33.3	0.0
Exercise 2	0.81	0.98	0.53	94.4	NA	0.0
Exercise 3	0.92	0.98	0.90	93.8	33.3	0.0
Exercise 4	0.89	0.95	0.95	100.0	50.0	16.7
Exercise 5	0.86	0.98	0.79	94.4	50.0	NA
Mean	0.88	0.97	0.75	96.5	41.7	4.2
95% CI low	0.82	0.96	0.51	92.6	26.4	0.0
95% CI high	0.95	0.99	0.98	100.0	57.0	17.4

**Table 4:** Probability of biopsy diagnosis given Gail model risk estimate and breast EIS result*

			Estimated outcome, %		
	Known evidence		Biopsy category		
Case frequency, %	EIS result	Gail cutoff'	Benign, no atypia	Infiltrating cancer or DCIS	Premalignant
74	Negative	Negative	91	3	7
16	Positive	Negative	65	9	26
7	Negative	Positive	53	27	19
3	Positive	Positive	18	45	37

*EIS indicates electrical impedance scanning; DCIS, ductal carcinoma in situ.
